# Transcriptome profiling and in silico detection of the antimicrobial peptides of red king crab *Paralithodes camtschaticus*

**DOI:** 10.1038/s41598-020-69126-4

**Published:** 2020-07-29

**Authors:** Igor A. Yakovlev, Erik Lysøe, Inger Heldal, Hege Steen, Snorre B. Hagen, Jihong Liu Clarke

**Affiliations:** NIBIO-Norwegian Institute of Bioeconomy Research, Aas, Norway

**Keywords:** Biotechnology, Molecular biology

## Abstract

Endogenous antimicrobial peptides (AMPs) are evolutionarily ancient factors of innate immunity, which are produced by all multicellular organisms and play a key role in their protection against infection. Red king crab (*Paralithodes camtschaticus*), also called Kamchatka crab, is widely distributed and the best known species of all king crabs belonging to the family *Lithodidae*. Despite their economic importance, the genetic resources of king crabs are scarcely known and no full-genome sequences are available to date. Therefore, analysis of the red king crab transcriptome and identification and characterization of its AMPs could potentially contribute to the development of novel antimicrobial drug candidates when antibiotic resistance has become a global health threat. In this study, we sequenced the *P*. *camtschaticus* transcriptomes from carapace, tail flap and leg tissues using an Illumina NGS platform. Libraries were systematically analyzed for gene expression profiles along with AMP prediction. By an in silico approach using public databases we defined 49 cDNAs encoding for AMP candidates belonging to diverse families and functional classes, including buforins, crustins, paralithocins, and ALFs (anti-lipopolysaccharide factors). We analyzed expression patterns of 27 AMP genes. The highest expression was found for Paralithocin 1 and Crustin 3, with more than 8,000 reads. Other paralithocins, ALFs, crustins and ubiquicidins were among medium expressed genes. This transcriptome data set and AMPs provide a solid baseline for further functional analysis in *P*. *camtschaticus*. Results from the current study contribute also to the future application of red king crab as a bio-resource in addition to its being a known seafood delicacy.

## Introduction

Endogenous antimicrobial peptides (AMPs) are evolutionarily ancient factors of innate immunity of multicellular organisms, and play a key role in their protection against infection^1,2^. AMPs were commonly considered to be a primitive mechanism of immunity and have been extensively studied in insects and other non-vertebrate organisms. There is now increasing evidence that AMPs play a crucial role in human immunity as well^3,4^. AMPs similar in structure and protective functions have been isolated from tissues of invertebrates^5,6^ and vertebrates^4,7^, as well as plants^8,9^.

Due to the lack of adaptive immunity which is present in vertebrates, invertebrates use relatively simple defense strategies that mainly rely on AMPs and innate immunity mechanisms, such as protective barriers, toxic molecules, and phagocytic cells that ingest and destroy invading microorganisms and larger parasites (such as worms)^10^.

Natural AMPs are generally small peptides up to 150 aa, with a molecular weight of 2 to 9 kDa, and containing a high portion of hydrophobic amino acids. AMP-encoding genes are either constitutively expressed or rapidly transcribed upon induction in eukaryotes by invading microbes and their products. These peptides are classified into families with distinct properties based on their amino acid sequences, number of cysteine residues, and spacing^9^.

Along with direct antimicrobial action, AMPs are able to participate in the regulation of immune processes and tissue regeneration^11^. Fundamental studies of AMPs are closely related to their important applied value. Natural peptides can become prototypes of new broad-spectrum antibiotic candidates which are urgently needed to tackle the challenge of antimicrobial resistance, a serious threat globally. The development of resistance to AMPs is rather difficult, since it requires major changes in the structure and electrophysiological properties of the cell membrane^12^. AMPs increase the permeability of the cell membrane, thereby enhancing the effect of traditionally used antibiotics and can be used in combination with them^11,13^.

According to the APD database (https://aps.unmc.edu/AP), about 3,073 natural AMPs have been discovered to date, and at least a third of them are from invertebrates^14^. Especially marine invertebrates could be a valuable source of new AMPs.

The red king crab (*Paralithodes camtschaticus*), also called Kamchatka crab or Alaskan king crab, is the best known species of all king crabs. It is the largest arthropod and has a crab-like morphology and a strong calcified exoskeleton with spines^15^, and belongs to the family *Lithodidae* (Anomura). The red king crab is widely distributed and native to the Bering Sea, around the Kamchatka Peninsula and neighboring Alaskan waters, and is also present in the Barents Sea (www.wikipedia.org). It became established in the latter in the mid-1990s after introduction to the area in the 1960s^16^. The red king crab is a highly valued delicacy on the international market and currently contributes significantly to the income from fisheries in the regions where it is harvested^17,18^.

In addition to its being a seafood delicacy with high demand and consumed globally, red king crab has other unexploited potential value as a source of new AMPs from a cold-water marine organism. To date only limited studies have been reported^19,20^. Moreover, the genetic resources of red king crabs are scarcely known^21^ and full-genome sequences are unknown, strongly suggesting that efforts should be given to the investigation of red king crab to reveal its potentially valuable genes, for both fundamental understanding and developing the bio-based economy. In this study, we sequenced the transcriptomes of various parts of *P*. *camtschaticus* (top shell, soft belly tissue, leg meat and leg shells), using Illumina high throughput sequencing technology. Sequences were submitted to the NCBI GeneBank (GHGY00000000.1). Bioinformatic analysis focused on putative AMPs with complete coding sequences (cds), and among these were three paralithocin genes with antimicrobial properties, defined and described recently^20^. Further identification of novel AMPs from red king crab could be of potential importance for extending the list of AMPs and contributing to future solutions for combating antimicrobial resistance. This transcriptome data set and AMPs provide a solid baseline for further functional analysis of *P*. *camtschaticus*.

## Results and discussion

### Transcriptome sequencing and assembly

The de novo transcriptomes of four types of tissues of two individuals of the red king crab *P. camtschaticus* (top shell, soft belly tissue, leg meat and leg shells) were sequenced on Illumina HiSeq 3/4,000 using two lanes and 2 × 150 bp (paired-end). This resulted in a draft gene set of 384,854 transcripts and 274,917 uni-genes of red king crab. Additionally, Augustus v3.3.2 was used to find full length transcripts. The uni-genes were annotated using a combination of blastx to several databases, such as nr, TrEMBL and Swiss-prot, and id mapped to eggnog, Kegg and GO. Distribution of GO terms of the red king crab biological processes, molecular functions and cellular compartments is presented in supplementary figure S1.

### In silico identification of AMPs

We used full length transcripts from red king crab and searched for similarity to putative AMPs, and found 30 AMP candidates belonging to diverse families and functional classes. For 27 of the predicted proteins we found full length gene models and for 7 just fragments, without 3′ or 5′ ends. Additionally, we found 19 considerably longer gene models (up to 2 kB), containing sequence motifs similar to known AMPs, often in several copies. Predicted in silico antimicrobial peptides of red king crab are presented in Table 1 and a full description is presented in Table S1.

**Table 1:** Predicted in silico antimicrobial peptides of Red King Crab.

Crab gene ID	Crab contigs	AMP*	Peptide length, aa		Comments	Annotation
PcBuf1	TRINITY_DN14568_c0_g1_i1	AP00308_Buforin	136			Buforin-like (histone H2A)/AP00489_Hipposin
PcBuf2	TRINITY_DN13_c0_g1_i8	AP00308_Buforin	124			Buforin-like (histone H2A)/AP00489_Hipposin
PcBuf3	TRINITY_DN118338_c0_g1_i1	AP02813_Acipensin	65	of 124	3′ fragment	histone H2A-like [*Osmia bicornis bicornis*]
PcAcp1	TRINITY_DN78095_c0_g1_i1	AP02811_Acipensin	97		fragment	Acipensin 1 (Ac1, bony fish, animals, XXE; UCLL1)
PcAcp2	TRINITY_DN80224_c0_g1_i1	AP02811_Acipensin	92		fragment	Acipensin 1 (Ac1, bony fish, animals, XXE; UCLL1)
PcAcp3	TRINITY_DN70256_c0_g1_i1	AP02813_Acipensin	126	of 138	5′ fragment	histone H2A [*Tetrahymena thermophila* SB210]
PcAcp4	TRINITY_DN106707_c0_g1_i1	AP02813_Acipensin	264	of 401	5′ fragment	core histone macro-H2A.1-like [*Penaeus vannamei*]
PcUbi1	TRINITY_DN95458_c0_g1_i1	AP02096_Ubiquicidin	131			Ubiquicidin (UBI 1–59, the ribosomal protein S30, human, primates, mammals, animals)
PcUbi2	TRINITY_DN26532_c3_g1_i1-RC	AP02096_Ubiquicidin	133			Ubiquicidin (UBI 1–59, the ribosomal protein S30, human, primates, mammals, animals)
PcLys1	TRINITY_DN4719_c0_g1_i3	AP02766_S. scrofa lysozyme	155		SigP	*S. scrofa* lysozyme (SSL; pigs, mammals; animals; UCSS1a; Derivatives: LP and SP)
PcßThm	TRINITY_DN201_c0_g1_i2	AP02533_cgTbeta	90			beta- thymosin 3 [*Penaeus japonicus*]
PcCrs1	TRINITY_DN1067_c1_g1_i1	AP02625_CqCrs	71		3′ fragment	CqCrs (CqCrustin, crustaceans, arthropods, invertebrates, animals; UCSS1a)/crustin antimicrobial peptide [*Portunus trituberculatus*]
PcCrs2	TRINITY_DN17819_c0_g2_i4	AP02625_CqCrs	132		SigP	CqCrs (CqCrustin, crustaceans, arthropods, invertebrates, animals; UCSS1a)/carcinin-like protein [*Penaeus vannamei*]
PcCrs3	TRINITY_DN2629_c0_g1_i6	AP02625_CqCrs	96		SigP	CqCrs (CqCrustin, crustaceans, arthropods, invertebrates, animals; UCSS1a)/crustin antimicrobial peptide [*Portunus trituberculatus*]
PcCrs4	TRINITY_DN592_c0_g3_i1	AP02625_CqCrs	140		3′ fragment	CqCrs (CqCrustin, crustaceans, arthropods, invertebrates, animals; UCSS1a)/crustin antimicrobial peptide [*Portunus trituberculatus*]
PcCrs5	TRINITY_DN592_c0_g4_i2	AP01555_CrusEs	105		SigP	CrusEs (*E. sinensis* Crustin, crustaceans, arthropods, invertebrates, animals)
PcCrs6	TRINITY_DN7452_c0_g1_i1	AP02752_CrustinPm1	178		SigP	CrusEs (crustin)/CrustinPm1 (UCSS1a; ?S = S; modular design; shrimp; crustaceans, arthropods, invertebrates, animals; BBL)/crustin 4 [*Panulirus japonicus*]
PcCrs7	TRINITY_DN8115_c0_g1_i5 + TRINITY_DN2054_c0_g1_i2	AP02625_CqCrs	103		SigP	CqCrs (CqCrustin, crustaceans, arthropods, invertebrates, animals; UCSS1a)/crustin antimicrobial peptide [*Portunus trituberculatus*]
PcCrs8	TRINITY_DN788_c0_g2_i2	AP02753_CrustinPm7	117		SigP	CrustinPm7 (UCSS1a; ?S = S; modular design; shrimp, crustaceans, arthropods, invertebrates, animals; BBL)
PcPar1	Newly assembled TRINITY_DN18_c0_g1_i2 + TRINITY_DN349_c20_g1_i2	AP02959_Paralithocin 1	60		SigP	Paralithocin 1 (the red king crab, crustaceans, arthropods, invertebrates, animals; 4S = S; UCSS1a) / MF919584
PcPar2	Newly assembled TRINITY_DN1423_c0_g1_i2 + TRINITY_DN26637_c1_g2_i2	AP02960_Paralithocin 2	72		SigP	Paralithocin 2 (the red king crab, crustaceans, arthropods, invertebrates, animals; 4S = S; UCSS1a) / MF919585
PcPar2.2	Newly assembled TRINITY_DN18_c0_g1_i4 + TRINITY_DN40550_c0_g1_i1	AP02960_Paralithocin 2.2	73		SigP	Paralithocin 2 (the red king crab, crustaceans, arthropods, invertebrates, animals; 4S = S; UCSS1a) / MF919585
PcPar3	TRINITY_DN5503_c0_g1_i1	AP02961_Paralithocin 3	77		SigP	Paralithocin 3 (the red king crab, crustaceans, arthropods, invertebrates, animals; 4S = S; UCSS1a) / MF919586
PcPar4	TRINITY_DN719_c0_g2_i4	AP00208_Peptide 3,910	62			Peptide 3,910/enhancer of rudimentary homolog isoform X2 [*Penaeus vannamei*]
PcKaz1	TRINITY_DN11095_c2_g1_i1	AP03038_SPINK9-v1	65			Proteinase inhibitor PSKP-1/AP03038—SPINK9-v1/Kazal-type serine proteinase inhibitor 1 [*Apostichopus japonicus*]/ Kazal 1&2 domains
PcKaz2	TRINITY_DN316_c0_g1_i3	AP03038_SPINK9-v1	65		SigP	SPINK9-v1/Kazal-type serine proteinase inhibitor 1 [Apostichopus japonicus]/5 Kazal 1 domains
PcALF1	TRINITY_DN20295_c2_g1_i1	AP02147_ALF	132		SigP	ALFpm3 (ALF-Pm3; anti-lipopolysaccharide factor)
PcALF2	TRINITY_DN3350_c1_g6_i1	AP02147_ALF	135		SigP	ALFpm3 (ALF-Pm3; anti-lipopolysaccharide factor)
PcALF3	TRINITY_DN435_c4_g1_i3	AP02147_ALF	126		SigP	ALFpm3 (ALF-Pm3; anti-lipopolysaccharide factor)
PcALF4	TRINITY_DN77684_c0_g1_i1	AP02147_ALF	123		SigP	ALFpm3 (ALF-Pm3; anti-lipopolysaccharide factor)
AMPs are part of the larger gene						
	RINITY_DN2036_c0_g2_i5	AP02012_YFGAP	334			YFGAP (Yellowfin Tuna GAPDH-related Antimicrobial Peptide; fish, animals)/glyceraldehyde 3-phosphate dehydrogenase [*Acartia pacifica*]
	TRINITY_DN142437_c0_g1_i1	AP02388_BPTI	105			BPTI (Bovine Pancreatic Trypsin Inhibitor; UCSS1a; cattle, ruminants, mammals, animals; 3S = S)
	TRINITY_DN167115_c0_g1_i1	AP02388_BPTI	83			BPTI (Bovine Pancreatic Trypsin Inhibitor; UCSS1a; cattle, ruminants, mammals, animals; 3S = S)
	TRINITY_DN20694_c0_g1_i5	AP02388_BPTI	821			BPTI (Bovine Pancreatic Trypsin Inhibitor; UCSS1a; cattle, ruminants, mammals, animals; 3S = S)
	TRINITY_DN22010_c0_g1_i3	AP02388_BPTI	61			BPTI (Bovine Pancreatic Trypsin Inhibitor; UCSS1a; cattle, ruminants, mammals, animals; 3S = S)
	TRINITY_DN25141_c0_g1_i6	AP02388_BPTI	764			BPTI (Bovine Pancreatic Trypsin Inhibitor)/papilin-like [*Hyalella azteca*]—11 Kinutz-BPTI domains
	TRINITY_DN27023_c0_g1_i2	AP02388_BPTI	103			BPTI (Bovine Pancreatic Trypsin Inhibitor; UCSS1a; cattle, ruminants, mammals, animals; 3S = S)
	TRINITY_DN627_c0_g1_i1	AP02388_BPTI	1,148			BPTI (Bovine Pancreatic Trypsin Inhibitor; UCSS1a; cattle, ruminants, mammals, animals; 3S = S)
	TRINITY_DN953_c0_g1_i14	AP02388_BPTI	2,318			BPTI (Bovine Pancreatic Trypsin Inhibitor; UCSS1a; cattle, ruminants, mammals, animals; 3S = S)
	TRINITY_DN2174_c0_g1_i1	AP02453_Scolopendin	303			Scolopendin 1 (myriapods, arthropods, invertebrates, animals; XXA)/cyclin-dependent kinase 1-like [*Penaeus vannamei*]
	TRINITY_DN106471_c0_g1_i1	AP02453_Scolopendin	79		fragment	Scolopendin 1 (myriapods, arthropods, invertebrates, animals; XXA)
	TRINITY_DN1514_c3_g5_i1	AP02453_Scolopendin	300			Scolopendin 1 (myriapods, arthropods, invertebrates, animals; XXA)/cyclin-dependent kinase 1-like [*Penaeus vannamei*]
	TRINITY_DN160752_c0_g1_i1	AP02453_Scolopendin	68		fragment	Scolopendin 1 (myriapods, arthropods, invertebrates, animals; XXA)
	TRINITY_DN2339_c0_g5_i1	AP02453_Scolopendin	297			Scolopendin 1 (myriapods, arthropods, invertebrates, animals; XXA)/cyclin-dependent kinase 1-like [*Penaeus vannamei*]
	TRINITY_DN92735_c3_g1_i1	AP02595_CcAMP1	295			CcAMP1 (insects, arthropods, invertebrates, animals)/medium-chain specific acyl-CoA dehydrogenase, mitochondrial isoform X3 [*Strigops habroptila*]
	TRINITY_DN2036_c0_g2_i3	AP02680_SJGAP	334		12 SapA and SapB domains	SJGAP (Skipjack Tuna GAPDH-related AMP, fish, animals, UCLL1a)/glyceraldehyde 3-phosphate dehydrogenase [*Eriocheir sinensis*]
	TRINITY_DN5804_c5_g1_i1	AP02712_Lc-NK-lysin	722		fragment	Lc-NK-lysin (fish, animals, 4S = S, UCSS1)/prosaposin-like [*Penaeus vannamei*]
	TRINITY_DN90639_c0_g1_i1	AP02797_cOT2	818		fragment	cOT2 (UCSS1; Siamese crocodile, reptiles, animals)/Transferrin [*Penaeus vannamei*]
	TRINITY_DN11894_c0_g1_i1	AP02797_cOT3	1,378			cOT2 (UCSS1; Siamese crocodile, reptiles, animals)/Transferrin [*Penaeus vannamei*]

*The antimicrobial peptide database (APD3)^14^.

Below we will characterize the most interesting groups of AMPs in red king crab.

**Figure 1:**
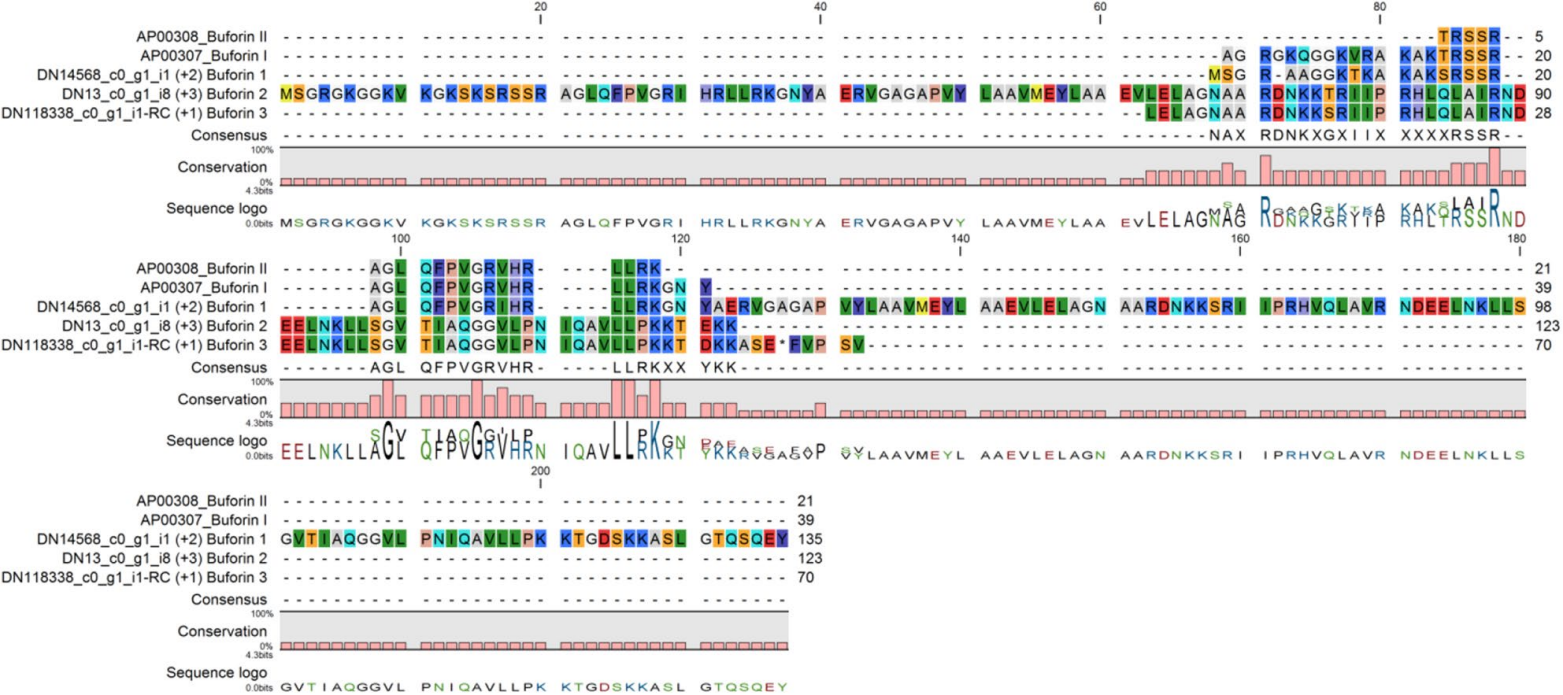
Alignments of the red king crab buforins with published data, Buforin 1 (AP00307) and Buforin II (AP00308).

Buforins are a large group of AMPs derived from the N-terminal region of histone H2A that interacts directly with nucleic acids. Buforin I is generated from histone H2A by pepsin-directed proteolysis in the cytoplasm of gastric gland cells and provides an antimicrobial protection in the stomach of the Asian toad *Bufo gargarizans*^22^. Natural buforin could be further treated by endoproteinase Lys-C to produce truncated versions of AMPs (21 aa) with higher antimicrobial activity. Buforins, which house a helix-hinge-helix domain, kill a microorganism by entering the cell without membrane permeabilization and thus binding to nucleic acids. The proline hinge is crucial for the cell penetrating activity of buforins^22,23^. We found three gene models encoding for buforin-like AMPs with high similarity to the toad’s Buforins I and II (Fig. 1).

There are two other groups of AMPs derived from histone H2A with sequence similarities to buforin—acipensins, isolated from leukocytes of the Russian sturgeon *Acipenser gueldenstaedtii*^24^, and hipposins, isolated from Atlantic halibut^25,26^. We found ten contigs encoding proteins similar to histone H2A and to buforin or acipensin AMPs. Two of them were more similar to Buforin 1 (AP00308) and 8 to acipensins (AP02813). Most acipensins were not full-length proteins but fragments (Table S1).

Another group of AMPs is ubiquicidins, a cytosolic antimicrobial protein that is identical or highly homologous to the ribosomal protein S30, where the precursor element shows homology to ubiquitin^27^. It preferentially binds to bacterial cell membrane at the site of infection^28^. There were two ubiquicidins with ORF length around 130 aa found in red king crab transcriptome.

Lysozyme is a naturally occurring enzyme found in bodily secretions such as tears, saliva, and milk. It functions as an antimicrobial agent by cleaving the peptidoglycan component of bacterial cell walls, which leads to cell death^29^. Due to the distinct antimicrobial properties, lysozyme has been used effectively in the food industry^30^. We found two contigs, one short and one long, containing a lysozyme gene with full length ORF and an active region of 131 aa. Similarly, lysozyme encoding genes named lys1 and lys2 are also characterized in nematodes (Clarke et al. ongoing work/unpublished data).

**Figure 2:**
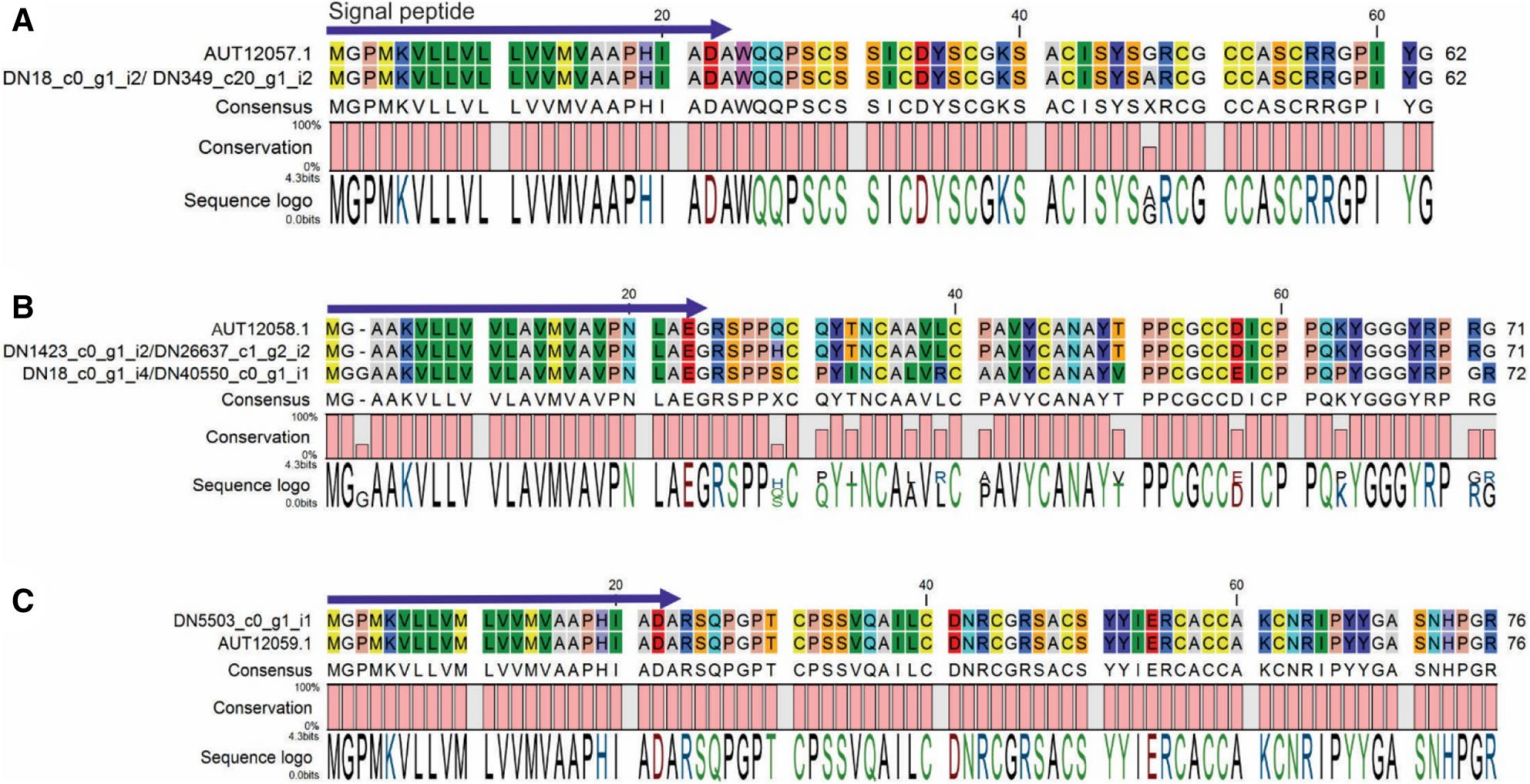
Alignments of the red king crab paralithocins from transcriptome data with published data: (**A**) Paralithocin 1—AUT12057.1; (**B**) Paralithocin 2—AUT12058.1; (**C**) Paralithocin 3—AUT12059.1.

Crab Paralithocins 1–3 were assigned to a previously unknown family of Cys-rich antimicrobial peptides with limited antimicrobial functions against marine bacteria^20^. The full length sequence of Paralithocin 1 was obtained by joining TRINITY_DN18_c0_g2_i4 and TRINITY_DN349_c20_g1_i2, with ORF found at positions 85 – 264. The full length protein has a size of 62 aa and contains a signal peptide (MGPMKVLLVLLVVMVAAPHIADA) with cleavage position at 23–24 aa (ADA-WQ). There is one aa substitution in comparison with published data (Fig. 1). Full length sequence of Paralithocin 2 was obtained by joining TRINITY_DN26637_c1_g2_i2 and TRINITY_DN1423_c0_g1_i2, with ORF found at positions 148 – 363. The full length protein has a size of 72 aa and contains a signal peptide (MGAAKVLLVVLAVMVAVPNLAEG) with cleavage position at 23–24 aa (AEG-RS). There is one aa substitution at position 5 in comparison with published data (Fig. 2). We detected another AMP very similar to Paralithocin 2, which we called Par2.2. The full length sequence of Paralithocin 3 was obtained from TRINITY_DN5503_c0_g1_i1, with ORF found at positions 59–289. The full length protein has a size of 77 aa and contains a signal peptide (MGPMKVLLVMLVVMVAAPHIADA) with cleavage position at 23–24 aa (ADA-RS) (Fig. 2B). No differences between predicted and published data were found for Paralithocin 3 (Fig. 2C).

Paralithocins 1 and 3 showed blast similarity (e − 4) to a protein with antimicrobial functions Mytilin (ADC29474, ADC29471) from sea mussel *(Mytilus coruscus*). Paralithocin 2 showed weak blast similarity (e − 3) to defensin proteins.

**Figure 3:**
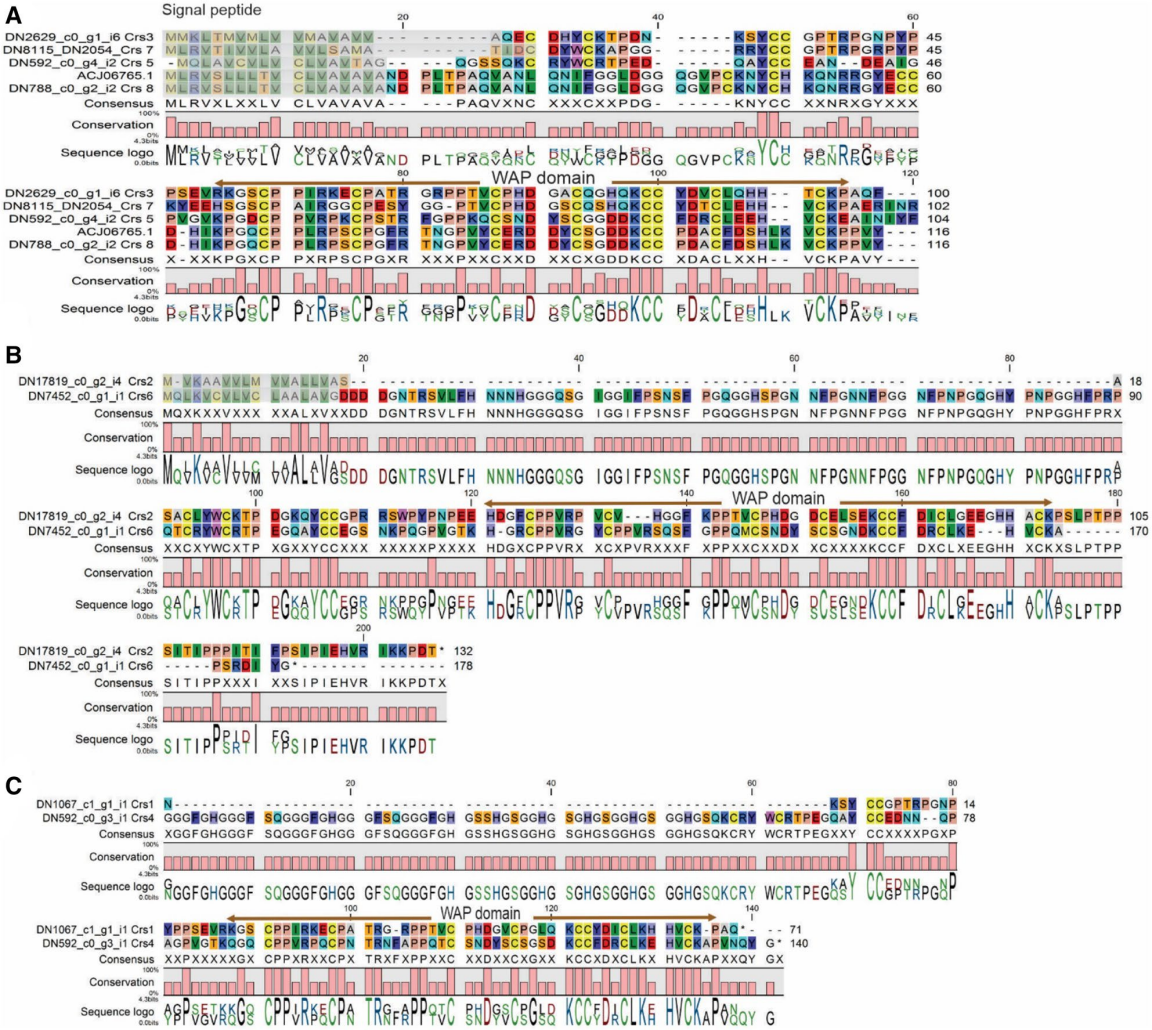
Alignments of the red king crab crustin sequences from transcriptome data against published crustin ACJ06765.1 (**A**). Crustins are grouped by their length and similarity: (**A**) Crustins 3, 5, 7 and 8; (**B**) Crustins 2 and 6; (**C**) Crustins 1 and 4 (3′ fragments without signal peptide). Signal peptides shadowed with grey.

Crustins are cationic cysteine-rich AMPs with a leader/signal sequence at the N-terminus and single whey acidic protein (WAP) domain at the C-terminus, and are expressed by the circulating haemocytes of crustaceans^31^. Crustins from the haemocytes of black tiger shrimp (*P. monodon*)^31^ or from freshwater prawn *Macrobrachium rosenbergii*^32^ exhibit potent anti-bacterial activity against several gram-positive and gram-negative bacteria from the environment. A single crustin was detected earlier in the haemocytes of red king crabs together with spider crabs^19^. We found eight typical crustins in the red king crab transcriptome with signal peptides (except two 3′ fragments) and distinct WAP domain. They showed high similarity (e <  − 20) to crustins from other crabs and marine species (Fig. 3).

We found one protein with similarity to AMP from pigs—enhancer of rudimentary homolog (AP00208), called peptide 3,910 with antibacterial properties. It is a small protein (103 aa) containing an ER domain (PF01133) and antimicrobial region of 29 aa, defined here as Paralithocin 4.

Furthermore, we identified two proteins containing Kazal-type serine protease inhibitor domains (PF00050), similar to human skin-originated SPINK9 AMPs. SPINK9 is a member of the epidermal antimicrobial peptides for selective killing of *E. coli*, which might contribute to the innate barrier function of human skin^33^. One defined protein is relatively short (65 aa) and contains Kazal 1 and Kazal 2 domains. Another protein is large (325 aa), contains signal peptide and 5 Kazal 1 and Kazal 2 domains.

Anti-lipopolysaccharide factor (ALF) is a small protein with broad-spectrum antimicrobial activity against gram-negative and gram-positive bacteria, and filamentous fungi, which has potential application in disease control. ALF was originally identified from horseshoe crabs and recently found in several shrimp species^34,35^. Different ALFs have a conserved cluster of positively charged residues within their disulfide loop between two conserved cysteine residues, which is usually called lipopolysaccharide (LPS)-binding domain (DUF3254), and considered to be the vital functional domain^34,36^. We defined four ALFs in red king crab. All of them possess signal peptides and typical DUF3254 domain and are quite different in amino acid composition.

Sequences matching to AMPs are often just part of large ORF, which corresponds to a large protein coding gene, e.g. Scolopendin AMP protein (AP02453) found in nearly 30 contigs which contain ORFs of cyclin-dependent kinase 2—(e.g. TRINITY_DN2174_c0_g1_i1). Similarly, AP02797_cOT2 (29 aa) found in TRINITY_DN90639_c0_g1_i1 is a part of Transferrin protein (*Penaeus vannamei*) of 350 aa. Thus, short AMPs could be processed from longer peptides. Such genes were also considered as AMP candidates. We defined 19 such large proteins with similarities to YFGAP (Yellowfin Tuna GAPDH-related Antimicrobial Peptide), BPTI (Bovine Pancreatic Trypsin Inhibitor), Scolopendin 1, SJGAP (Skipjack Tuna GAPDH-related AMP), transferrins and some AMPs from trout. Active short AMPs could be produced by posttranslational processing, proteolysis or degradation of such longer peptides^4^, but this should be proved specifically. They are presented in Tables 1 and S1.

To evaluate the defined AMPs, we also ran a prediction tool using the CAMP_R3_ database^37^, confirming that most of the defined proteins are AMPs (Table S2). At the same time the putative AMP properties of PcPar4 (Paralithocin 4) and PcKaz1 (Kazal-type serine proteinase inhibitor 1), and of two large proteins Scolopendin 1 (DN160752_c0_g1_i1) and BPTI (DN22010_c0_g1_i3) are yet to be confirmed. The underlying reasons could be due to these peptides being novel or weak AMPs. Future study should focus on characterization of these AMPs.

### Analysis of selected AMP gene expression

**Figure 4:**
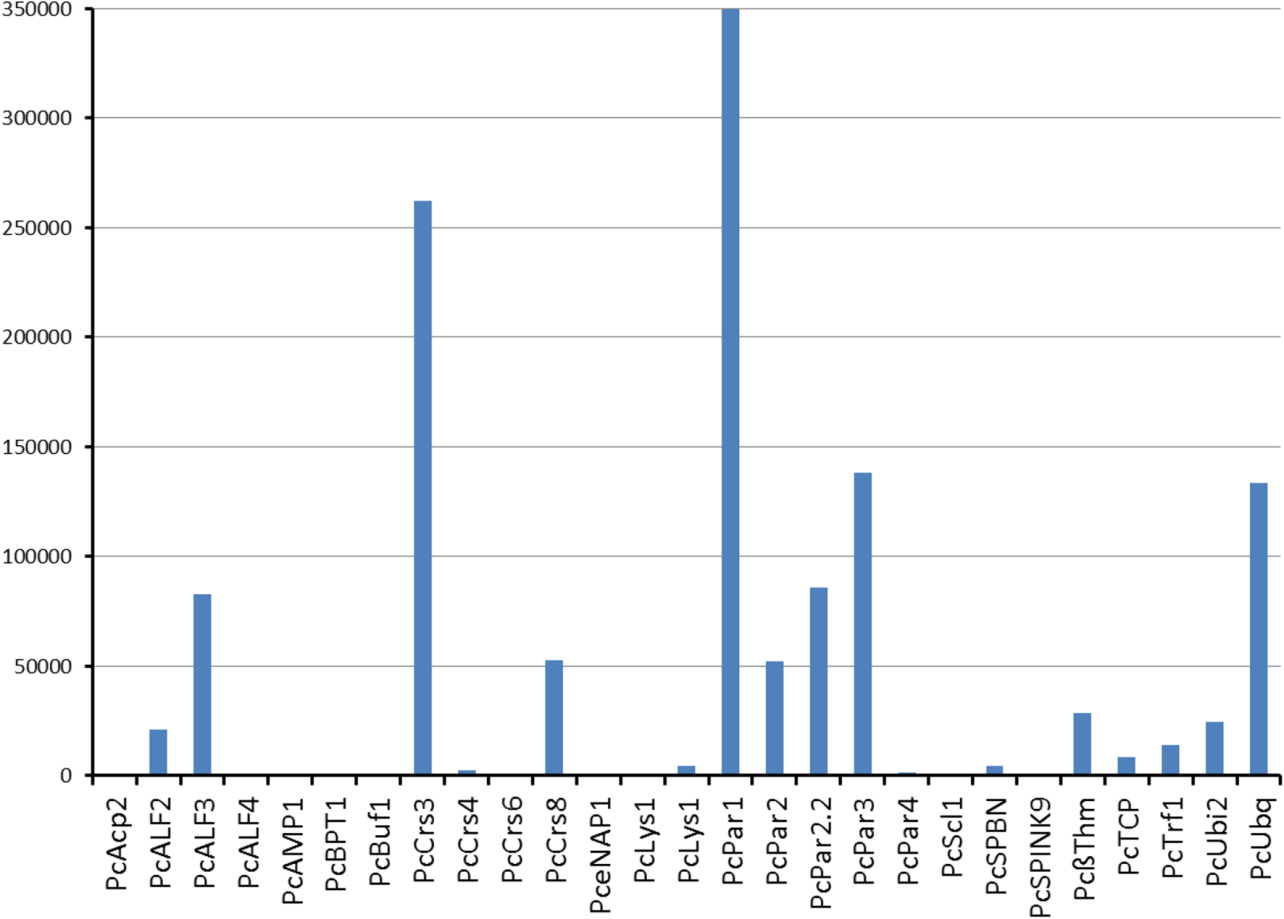
Average RPKM expression levels of some AMP genes in three independent king crabs with RNAs from carapace, tail flap, leg flesh, and legs containing both shell and flesh based on RNA-seq data.

Constitutive expression of selected candidate AMP encoding genes was conducted according to RNA-seq data (Table S3). We tested the RPKM expression of some AMP genes in three independent king crabs with RNAs from carapace, tail flap, leg flesh, and legs containing both shell and flesh (Fig. 4, Table 2). In total we analyzed expression patterns of 27 AMP genes. 19 genes were highly expressed with more than 3,000 reads in all libraries, 5 genes were expressed with around 1,000 reads, and 3 genes had less than 100 reads and were low-expressed. The highest expression was found for Paralithocin 1 and Crustin 3 with more than 8,000 reads. Other paralithocins, ALFs, crustins and ubiquicidins were among medium-expressed genes.

**Table 2:** Description of red king crab tissues used for RNA extraction.

Sample name	Description
B-top	Top shell of crab B
C-top	Top shell of crab C
A-belly	Soft tissue under crab A
A-legm	Meat inside the leg of crab A
C-legm	Meat inside the leg of crab C
A-legs	Leg meat and shell of crab A
C-legs	Leg meat and shell of crab C

## Conclusions

In conclusion, transcriptome profiling and in silico annotation was very effective to define candidate AMPs in the red king crab transcriptomes. Gene models assembled from transcriptome data will be a valuable resource for future studies, including functional annotation of the red king crab genome. In our study we identify a significant number of AMPs as candidates for future studies of antimicrobial activity. These candidate AMPs will be tested for production of recombinant enzymes in plants or algae and for antimicrobial activity against marine microorganisms (to substitute antibiotics or increase their efficiency for salmon production).

## Materials and methods

### DNA and RNA extraction

Three live adult red king crab individuals were kindly provided by Norway King Crab Ltd (https://nkc.no). They were stored in a freezer at − 80 °C before DNA and RNA isolation.

Total DNA was extracted from each sample with the DNeasy Blood & Tissue Kit (Qiagen, Cat No: 69504) according to the manufacturer’s instructions. The concentration and integrity of the RNA were assessed with a Thermo Scientific NanoDrop 8,000 Spectrophotometer and Agilent 2,100 Bioanalyzer, respectively (Agilent Technologies, USA).

RNA was extracted from four tissues from three adult individuals; see Table 2 for sample description. Total RNA was extracted from each sample with the Direct-zol RNA Miniprep Plus kit (Zymo, R2071) according to the manufacturer’s instructions after treatment with RNase-free DNase I (Qiagen) to eliminate genomic DNA. The concentration and integrity of the RNA were assessed with a Thermo Scientific NanoDrop 8,000 Spectrophotometer and Agilent 2,100 Bioanalyzer, respectively (Agilent Technologies, USA).

### Transcriptome sequencing and assembly

For library preparation ~ 1 μg of total RNA was used. Sequencing and library preparation were performed by the Norwegian Sequencing Centre (NSC) in Oslo.

The raw data were trimmed using trimmomatic v0.38. Clean reads were assembled de novo using Trinity v2.8.4^38^, and then redundancy was reduced using cd-hit-est (v 4.7). The resulting graft gene set and uni-genes were preliminarily annotated by looking for similarities to uni-genes in several databases, such as nr, TrEMBL and Swiss-prot, and id mapping to eggnog, Kegg and GO. Full length gene models of the Trinity transcripts were predicted using Augustus v3.3.2^39^.

RPKM was used to identify expression patterns of the selected AMP genes in the different tissues (Fig. 4).

### AMP prediction

For prediction of AMPs from transcriptomes of red king crab we used assembled contigs, full length gene models and AMP data from several databases. We screened our comprehensive transcriptome datasets using several public AMP databases and tools^14,40,41^. Among these was the Data Repository of Antimicrobial Peptides (DRAMP) updated to version 2.0, containing a total of 19,899 entries (newly added 2,550 entries), including 5,084 general entries, 14,739 patent entries, and 76 clinical entries^40^. The antimicrobial peptide database (APD3) recently contained 3,072 entries of AMPs^14^. In the integrated system for identifying Anti-Microbial Peptides (dbAMP) there are 12,389 unique entries, including 4,271 experimentally verified AMPs and 8,118 putative AMPs along with their functional activities, supported by 1924 research articles^41^. CAMP_R3_ (Collection of Anti-Microbial Peptides) contains information on the conserved sequence signatures captured as patterns and Hidden Markov Models (HMMs) in 1,386 AMPs represented by 45 families and tools for identification of antimicrobial peptides^37^.

We used two approaches to define AMP candidates. Initially we used protein sequences of obtained full length gene models to blastp (protein–protein BLAST) against AMP database data. This approach was not effective for very short AMP proteins.

In the second approach, we made a tblastn (protein–translated nucleotide sequence BLAST) search of AMPs from all four databases against assembled contigs translated into proteins by all six ORFs using CLC Genomics Workbench 10.0 (https://www.qiagenbioinformatics.com/). The tblastn results were filtered with a similarity score ≥ 90. Sequences with observed similarity at the given cutoff values were considered as AMP candidates. Finally, candidate AMP contigs were manually searched for ORFs containing continuous stretches of amino acids of described AMPs, for translation start and stop codons to define the full length or fragment protein sequences. Defined in such a way, proteins were further analyzed for the presence of signal peptides and functional domains. Similarity to known AMPs was additionally confirmed by blasp against the NCBI database and the AMP prediction tool of the CAMP_R3_ database^37^.

All four databases showed similar results, so finally we focused on the ADP3 database. Each AMP candidate was manually analyzed, and we discarded all low similarity and redundant AMPs (i.e. different AMPs matching to the same crab contigs).

## Supplementary information

Supplementary Figure S1

Supplementary Table S1

Supplementary Table S2

Supplementary Table S3

Supplementary Information
