# Supplementary figures and images for: Transcriptome profiling and in silico detection of the antimicrobial peptides of red king crab Paralithodes camtschaticus
Source: Sci Rep. 2020 Jul 29;10:12679. doi: 10.1038/s41598-020-69126-4 (PMC7391757; doi:10.1038/s41598-020-69126-4)

## Biological process

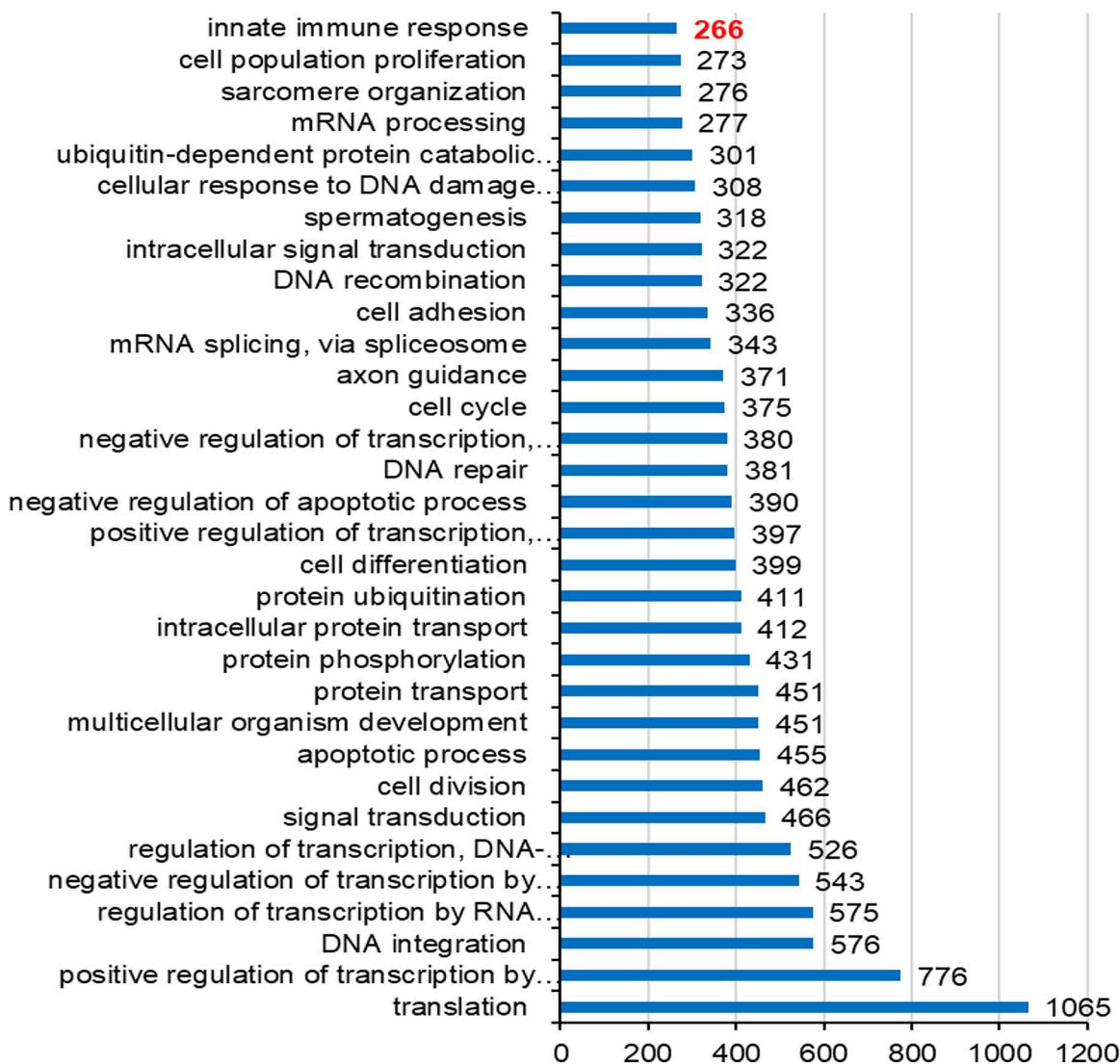

## Molecular function

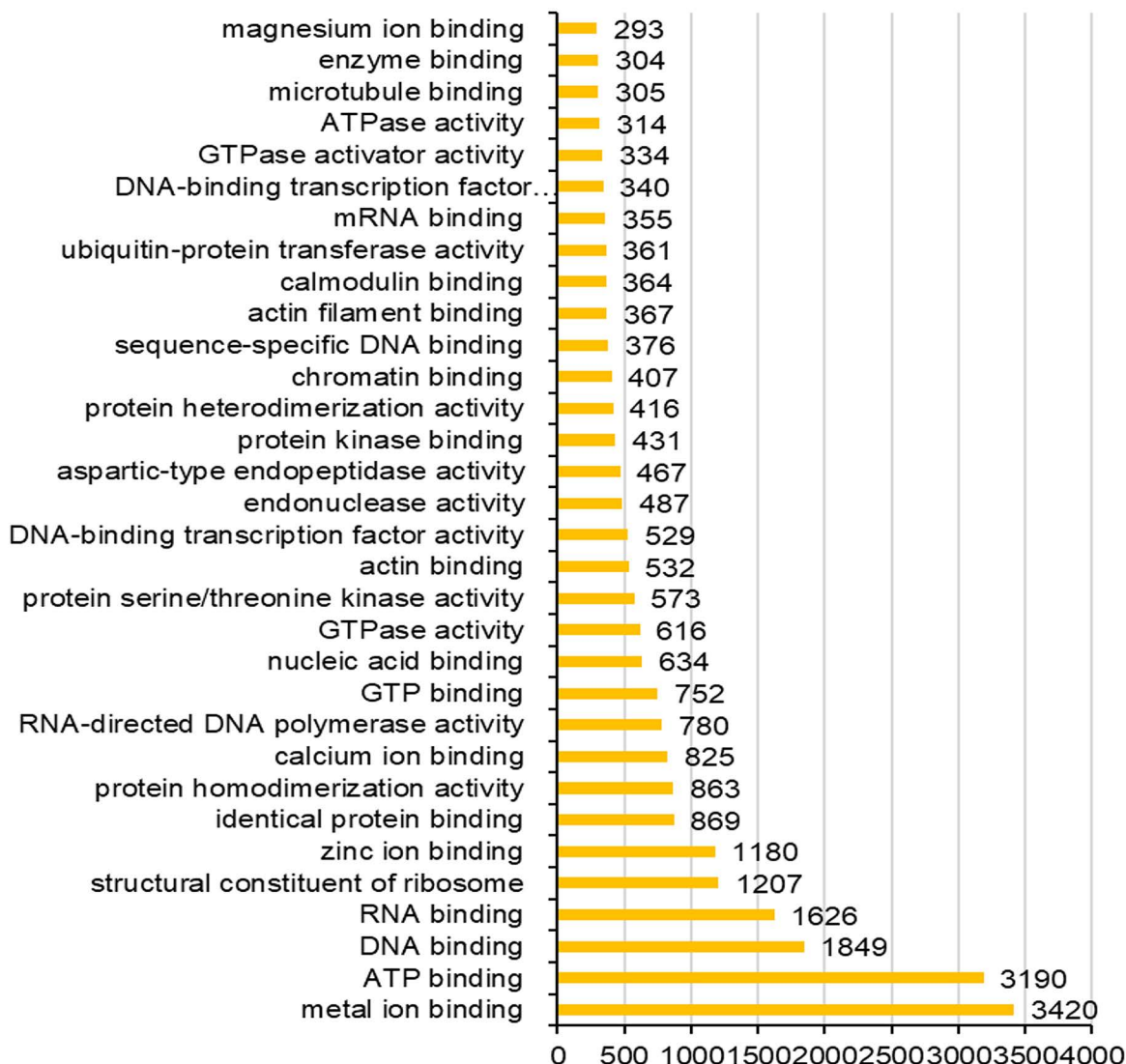

## Cellular components

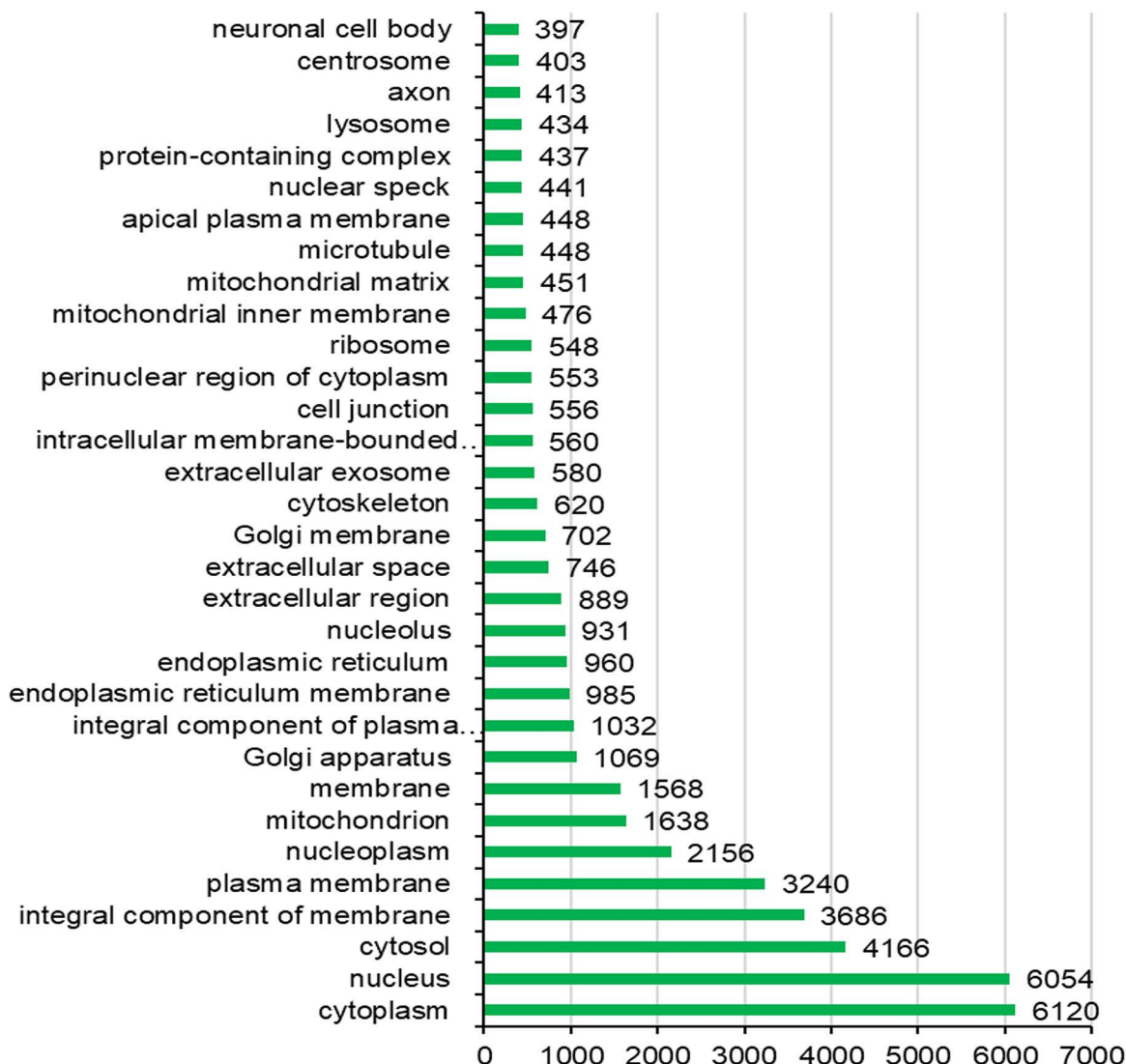

Supplement: Supplementary file 1 — Supplementary Figure S1 [file 41598_2020_69126_MOESM1_ESM.pdf]
