# Supplementary material for: Transcriptome profiling and in silico detection of the antimicrobial peptides of red king crab Paralithodes camtschaticus
Source: Sci Rep. 2020 Jul 29;10:12679. doi: 10.1038/s41598-020-69126-4 (PMC7391757; doi:10.1038/s41598-020-69126-4)
Supplement: Supplementary file 5 — Supplementary Information [file 41598_2020_69126_MOESM5_ESM.pdf]

Supplementary files to manuscript:

Transcriptome profiling and *in silico* detection of the antimicrobial peptides of red king crab *Paralithodes camtschaticus*

Igor A. Yakovlev[1], Erik Lysøe[1], Inger Heldal[1], Hege Steen[1], Snorre B. Hagen[1*], Jihong Liu Clarke[1*]

[1] NIBIO- Norwegian Institute of Bioeconomy Research, Aas, Norway

File 1: Manuscript_Red king crab_Table S1_candidate AMPs

File 2: Manuscript_Red king crab_Table S2_AMP analysis

File 3: Manuscript_Red king crab_Table S3_expression

File 4: Manuscript_Red king crab_Figure S1
